# Relapse-Fated Subclones: Finding the Needle in the Haystack

**DOI:** 10.1097/HS9.0000000000000374

**Published:** 2020-05-21

**Authors:** Melania Tesio

**Affiliations:** Laboratory of Onco-hematology, Institut Necker Enfants Malades (INEM), Institut national de Recherche Médicale (INSERM) U1151, Paris, France

In a recent *Cancer Discovery* paper, Stephanie Dobson et al identified rare relapse-fated subclones in B-ALL and showed that they possess an intrinsic drug tolerance as well as metabolic and stemness signatures. The identification of these subclones and associated pathways will be instrumental to target relapse-fated cells, thereby preventing their full evolution into relapse clones.

Clonal evolution is a major driver of disease progression and relapse in hematological malignancies. In this process, genetic and epigenetic mechanisms intervenient to shape leukemia composition, favoring the emergency of multiple co-existing leukemic clones, including those ultimately driving relapse. In acute lymphoblastic leukemia (ALL), where a branching evolution takes place, the clone driving relapse is not derived from a major diagnostic clone but rather from a minor ancestral one.^1^

In the attempt to understand how cytotoxic chemotherapy shapes clonal evolution and drug resistance, numerous studies have compared diagnosis and relapsed samples.^2–3^ Nevertheless, the molecular determinants that confer a diagnostic subclone a relapse fate before its evolution into a drug-resistant relapsing clone following chemotherapy exposure remain unclear.

In the recent *Cancer Discovery* paper, Dobson et al undertook a strategy which enabled the identification and further characterization of rare relapse-fated subclones.^4^ The authors performed whole exome sequencing of 14 paired diagnosis and relapse B-ALL patients samples encompassing distinct cytogenetic subtypes. This analysis allowed them to identify somatic single nucleotide variants, insertion-deletion mutations and DNA copy number alterations in bulk patient samples at diagnosis as well as at relapse. In parallel, diagnosis and relapsed samples were injected at limiting dilution doses in immunodeficient mice. In this way, the leukemic variants identified in the primary samples could be next target sequenced in the engrafted mice. By comparing the variant allele frequencies of the leukemic variants in diagnosis/relapse patient samples and xenografts, the authors were able to observe distinct clonal compositions in distinct xenografts which were issued from the same diagnostic sample. This indicates that the distinct leukemia-initiated cells from which these grafts originated were derived from genetically diverse subclones present at diagnosis. Hence, by performing large-scale xenograft assays at limiting dilution and combining them to genomic analysis, the researchers managed to capture a wide range of diagnostic subclones, which were endowed of distinct competitive repopulation capacity, immunophenotype, migration and proliferation capacities. Importantly, as evidenced by population phylogenetic analysis and mutation evolutionary trees, the diagnostic subclones captured by limiting dilution patient-derived xenografts (PDXs) included diagnosis relapse-initiating clones (dRI), namely very minor latent subclones present in the diagnostic samples and fated to relapse.

Why these clones were fated to relapse and other ones not? The experimental evidences provided by Dobson et al indicate that dRI possess at least 3 major features. The first one is an intrinsic drug tolerance. When compared to PDXs issued from major diagnostic clones, at least some of the dRI-derived PDXs analyzed showed reduced sensitivity to standard chemotherapy drugs, such as dexamethasone, vincristine and L-asparaginase. Hence, albeit this analysis included a limited number of samples, it suggests that drug tolerance arises prior chemotherapy exposure.

Second, dRI subclones possess a mitochondrial metabolic signature. Transcriptional analysis of diagnostic-derived PDXs and dRI-derived PDXs revealed that the latter is enriched with pathways involved in aminoacid metabolism, Krebs cycle, oxidative phosphorylation and lipid metabolism. Interestingly, these pathways were commonly enriched in dRI-derived PDX and relapse-derived PDX, suggesting that a metabolic rewiring underlies the road to relapse.

Last but not the least, hematopoietic stem cell genes, chromatin remodeling and cell stress responses signatures, such as unfolded protein responses, were also commonly enriched in dRI-derived PDX and relapse-derived PDX, suggesting their involvement in the evolution to relapse.

Taken together, these data suggest a model whereby relapse fate arises stochastically prior chemotherapy exposure from genetic and/or epigenetic mechanisms that contribute to rewire the cellular metabolism, confer stemness properties and modulate stress response pathways (Fig. 1). In this context, the chemotherapy pressure further promotes the evolution of dRI into relapse clones.

**Figure 1:**
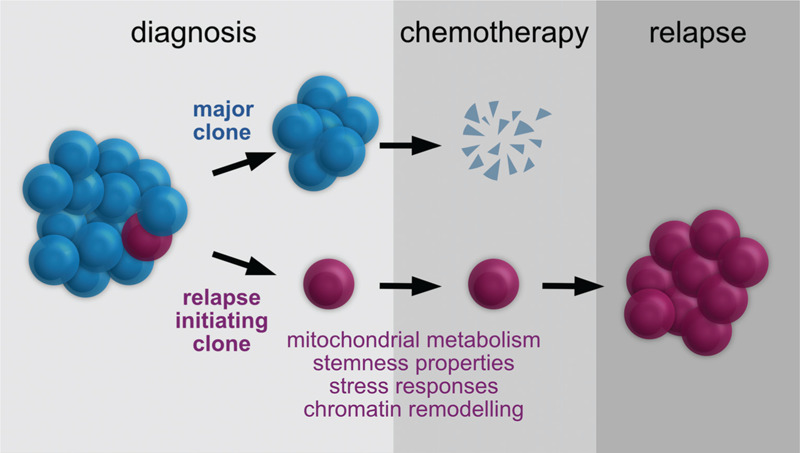
Diagnosis initiating-relapse subclones in the model proposed by Dobson et al.^4^.

How do the pathways identify shape the relapse fate? Stemness features might contribute to keep dRI dormant^5^ and/or facilitate their interaction with protective bone marrow niches, and/or to provide them with the self-renewal capacity to initiate relapse following dormancy. In addition, metabolic changes might be instrumental to provide drug tolerance and/or to enable them to be counter-selected during chemotherapy exposure.^6^ Similarly, stress responses might be necessary to guarantee the survival of dRI subclones following drug treatment. Further experiments will be necessary to fully elucidate these questions and to understand which genetic and/or epigenetic mechanism favors the emergency of dRI subclones. Nevertheless, the pathways identified by Dobson et al will be instrumental to target relapse-fated subclones, thereby preventing their full evolution to relapse.

In conclusion, thanks to a titanic experimental strategy, Dobson et al managed to a find the needle into the haystack; and of foremost, important therapeutic implications.

## References

[1] Mullighan CG Phillips LA Su X Genomic analysis of the clonal origins of relapsed acute lymphoblastic leukemia. *Science.* 2008;322:1377–1380. 19039135 10.1126/science.1164266 PMC2746051

[2] Ma X Edmonson M Yergeau D Rise and fall of subclones from diagnosis to relapse in pediatric B-acute lymphoblastic leukaemia. *Nat Commun.* 2015;6:6604. 25790293 10.1038/ncomms7604 PMC4377644

[3] Oshima K Khiabanian H da Silva-Almeida AC Mutational landscape, clonal evolution patterns, and role of RAS mutations in relapsed acute lymphoblastic leukemia. *Proc Natl Acad Sci U S A.* 2016;113:11306–11311. 27655895 10.1073/pnas.1608420113 PMC5056035

[4] Dobson SM, Garcia-Prat L, Vanner RJ, et al. Relapse fated latent diagnosis subclones in acute B lineage leukaemia are drug tolerant and possess distinct metabolic programs. *Cancer Discov*. 2020 February 21. [Epub ahead of print]. 10.1158/2159-8290.CD-19-1059 PMC7122013 32086311

[5] Ebinger S Özdemir EZ Ziegenhain C Characterization of rare, dormant, and therapy-resistant cells in acute lymphoblastic leukemia. *Cancer Cell.* 2016;30:849–862. 27916615 10.1016/j.ccell.2016.11.002 PMC5156313

[6] Burt R Dey A Aref S Activated stromal cells transfer mitochondria to rescue acute lymphoblastic leukemia cells from oxidative stress. *Blood.* 2019;134:1415–1429. 31501154 10.1182/blood.2019001398 PMC6856969

